# How to Establish and Follow up a Large Prospective Cohort Study in the 21st Century - Lessons from UK COSMOS

**DOI:** 10.1371/journal.pone.0131521

**Published:** 2015-07-06

**Authors:** Mireille B. Toledano, Rachel B. Smith, James P. Brook, Margaret Douglass, Paul Elliott

**Affiliations:** MRC-PHE Centre for Environment and Health, Department of Epidemiology and Biostatistics, School of Public Health, Imperial College London, St Mary’s Campus, London, United Kingdom; University of Oxford, UNITED KINGDOM

## Abstract

Large-scale prospective cohort studies are invaluable in epidemiology, but they are increasingly difficult and costly to establish and follow-up. More efficient methods for recruitment, data collection and follow-up are essential if such studies are to remain feasible with limited public and research funds. Here, we discuss how these challenges were addressed in the UK COSMOS cohort study where fixed budget and limited time frame necessitated new approaches to consent and recruitment between 2009-2012. Web-based e-consent and data collection should be considered in large scale observational studies, as they offer a streamlined experience which benefits both participants and researchers and save costs. Commercial providers of register and marketing data, smartphones, apps, email, social media, and the internet offer innovative possibilities for identifying, recruiting and following up cohorts. Using examples from UK COSMOS, this article sets out the dos and don’ts for today's cohort studies and provides a guide on how best to take advantage of new technologies and innovative methods to simplify logistics and minimise costs. Thus a more streamlined experience to the benefit of both research participants and researchers becomes achievable.

## Introduction

Cohort studies are typically expensive and time-consuming to establish and follow-up—these are commonly listed ‘weaknesses’ in epidemiology textbooks (e.g. Ward et al. [1]). This is partly because response rates in epidemiological studies have dropped dramatically over the past 60 years. In the early 1950s, the British Physicians Study and the Framingham Heart Study achieved response rates of 69% [2, 3]. The Nurses’ Health Study II achieved 24% in 1989 [4]. Today recruitment is even more challenging, e.g. 5.5% response rate for UK Biobank [5], whilst lower than expected recruitment rates in the US National Children’s Study have required expansion of study sites and revision of sampling plans [6]. Recruitment and questionnaire data collection in large cohorts have typically been via invitation letter and questionnaire by post. For large cohorts printing and mailing costs can be prohibitive, often requiring staggered recruitment to manage logistics of mass invitation mailings and returned consent forms/questionnaires. Baseline data collection may thus be drawn out over a long time period and funding bodies may be increasingly reluctant to commit resources to such an endeavour especially where response rates are low.

But do cohort studies need to be so expensive and time-consuming? We asked ourselves this when establishing the UK COSMOS cohort, having achieved lower than expected response rates in Phase 1 recruitment using established approaches. UK COSMOS is the UK arm of the international COSMOS study, a long-term prospective cohort study of possible health effects associated with use of mobile phones and related technology including nearly 300,000 adult mobile phone users across Europe [7]. With over 100,000 participants UK COSMOS is the largest component of the international cohort, the UK’s fourth largest cohort study, and the UK’s largest cohort study dedicated to environment and health. Baseline questionnaire data include use of mobile phones and other wireless technologies, other environmental exposures, health, lifestyle and demographic factors. Self-reported mobile phone use is supplemented by objective traffic data from network operators, and data on health events, e.g. cancer incidence, neurological diseases and mortality, is collected via tracking of disease/mortality registries and linkage to hospital episode data, where participants have consented to this. The international study is described in detail elsewhere [7].

Cohort studies may reduce costs and save time by finding efficiencies in the choice of: sampling population; invitation method; data collection; use of incentives, and use of reminders to increase response rates; and in follow-up methods. Here we discuss how these challenges were addressed in the context of our cohort study where fixed budget and limited time frame necessitated new approaches to consent and recruitment. This involved various innovative methods using new technologies during 7 recruitment phases (Table 1) and a total of ~3 million recruitment invitations between 2009–2012.

**Table 1:** Summary of Study Methods and Participation By Recruitment Phase of the UK COSMOS Study, 2009–2012.

Recruitment phase (and description)	1 (Pre-test)	2 (1st major campaign)	3 (SMS campaign)	5 (Electoral register pilot)	6 (Paid incentive pilot)	7 (2nd major campaign)	Total^a^
**Study methods**							
When	2009	**2010**	2011	2012	2012	**2012**	
Sampling frame	Mobile subscribers	**Mobile subscribers**	Direct marketing list	Edited electoral register	Edited electoral register	**Edited electoral register**	
Invitation method	Letter	**Letter**	SMS	Letter	Letter	**Letter**	
Consent + registration method	Paper	**Web**	Web	Web	Web	**Web**	
Questionnaire method	Paper or Web	**Web**	Web	Web	Web	**Web**	
Incentive used (if any)	None	**Prize draw (100 x £25)**	None	Prize draw (20 x £100)	£10 Gift voucher^b^	**£10 Gift voucher** ^c^	
Friends & Family statement in invitation	No	**No**	No	Yes	No	**Yes**	
**No. of invitations** ^d^	4,500	**2,395,500**	31,500	20,704	2,500	**645,000**	3,099,704
**No. of participants (N)**	244	**67,793**	42	399	181	**36,316**	105,028
**Recruitment rates (%)**	5.4	**2.8**	0.1	1.9	7.2	**5.6**	
**Time period (days)**	217	**130**	15	54	83	**124**	

Footnotes:

^a^ Total includes N = 53 additional volunteers who were recruited between Phase 3 and 5 (and chronologically collectively classed as Phase 4), in response to various recruitment strategies, including 2 participants recruited via a Facebook advert trial; these strategies were run concurrently and response rates cannot be calculated for comparisons, therefore are not shown in detail here.

^b^ Gift voucher offer in Phase 6 ceased at Day 25 (17/06/2012).

^c^ Gift voucher offer in Phase 7 ceased at Day 20 (05/09/2012), as recruitment target of 100,000 reached.

^d^ Number of invitations actually received, opened and read may be lower, e.g. if invitation is returned to Sender

## Methods

The protocol of the UK COSMOS study, and subsequent amendments were approved by the North West Haydock Research Ethics Committee (ref 08/H1010/90). Participants gave written electronic informed consent before taking part in the study.

### Choice of sampling population

Initially we sampled from mobile subscriber lists (stratified sampling by call time, age and sex in order to maximise exposure contrasts), then from a commercial direct marketing list and the UK Edited Electoral Register (Table 1).

Sampling from mobile subscriber lists provided a large sample (2.4 million mobile subscribers). However, agreeing data provision contracts with UK network operators in order to ensure confidentiality of subscriber details was long (7 years) and complex.

Direct marketing lists are compiled from various sources (e.g. electoral roll, online purchases, surveys); and can offer millions of records, with potential for targeted sampling via different contact routes (e.g. post, email, telephone, SMS). Provision of direct marketing data was rapid (~48 hours) and efficient, but comes at a (significant) cost.

The Edited Electoral Register is a large source of name and address data for adults registered to vote in the UK, available for purchase. Purchasing via a commercial provider was the easiest way to access these data on the national scale. UK electoral register data were rapidly available, relatively inexpensive and cheaper than enhanced direct marketing data, however study invitation method was limited to post.

### Invitation method

Prior to recruitment we undertook six rounds of focus groups across the UK with different socioeconomic, age, gender, and ethnic groups to inform study materials, and thus boost response rates. Initially we invited 2.4 million mobile subscribers via mail. The invitation pack (containing a letter from the mobile operator, a letter from Imperial College and a study information leaflet) was sent by the subscriber’s mobile operator on behalf of Imperial College to ensure customer confidentiality and comply with Data Protection law. Printing and mailing costs were a large proportion of our budget, so we looked for alternative invitation methods. Invitations in subsequent phases were sent direct from Imperial College as they did not rely on sampling from mobile subscriber lists. As cost per SMS is negligible compared with a mailed invitation, we conducted a pilot study in 2011 to evaluate efficacy of SMS invitations to participate in our cohort. 31,500 mobile phone users who had opted-in to receive both SMS and email marketing were selected from a direct marketing list. Individuals were randomly assigned to receive either initial SMS followed by reminder SMS (Group 1) or initial SMS followed by reminder e-mail (Group 2). We did not test email alone because we judged SMS to be a more appropriate route to reach mobile phone users and that the likelihood of people opening and reading an SMS, even from a number they did not recognise, would be high; whereas high levels of spam email mean that people often filter unsolicited emails from senders they do not recognise without reading them. We also trialled an invitation advert on Facebook for four weeks in 2012, which displayed to Facebook users based on their age and location (i.e. 18 or over, located in the UK).

Large-scale recruitment campaigns require resources in place to deal with queries from potential participants. For Phase 1 and 2 we contracted call handling to an external call centre provider, whilst queries by email and letter were dealt with in-house by the research team. There was a protocol in place to rapidly escalate any adverse reactions to the principal investigator. Once call volume had reduced, we brought call handling in-house to be dealt with by the research team. When call/email volume was predicted to increase (i.e. the second major recruitment campaign in 2012) we set up a formal call centre in-house. We designed and implemented in-house call centre management software that enabled the recording of call data and managing escalation workflow.

### Data collection

In Phase 1 participants returned a paper consent form, and could complete either a paper or web-based baseline questionnaire. For all subsequent phases, we used a fully web-based recruitment and data collection process comprising signatureless electronic consent (e-consent) form, registration form and questionnaire (Table 1). We obtained National Research Ethics Service approval for this approach. On receipt of a study invitation, participants visited the study website, logged in with their unique identifier (UID), completed e-consent, registration, and created a password. Participants logged into the baseline questionnaire using their secure UID and password combination. Save and return options were available on the questionnaire, but for security and confidentiality, consent and personal details were not accessible once submitted.

### Use of incentives

We used a variety of prize draw and gift voucher incentives (Table 1) to increase recruitment. In phases 5, 6 and 7 we took advantage of the ‘wider appeal’ of a gift voucher incentive and did not limit participation to named addressees (any eligible volunteer could participate, and spin-off recruitment through family and friends was encouraged in invitation letters sent in Phases 5 and 7) (Table 1).

### Using reminders

We sent reminder letters to increase response rate in recruitment campaigns, and we used email and SMS reminders to encourage questionnaire completion amongst participants who had not completed in a single session.

### Direct follow-up with participants

Email addresses were captured at recruitment from Phase 2 onward, as an essential requirement. As email is far cheaper than printing/mailing, we have sent e-newsletters to our cohort annually. We built a secure web-based ‘update portal’ requiring UID and password login on our study website, which allows participants to update contact details quickly integrating new information with existing databases. We send an annual email request asking participants to confirm/update their details. We have used both Facebook and Twitter to provide study updates and link to relevant news stories to maintain long-term interest in the study and research question.

## Results

**Fig 1:**
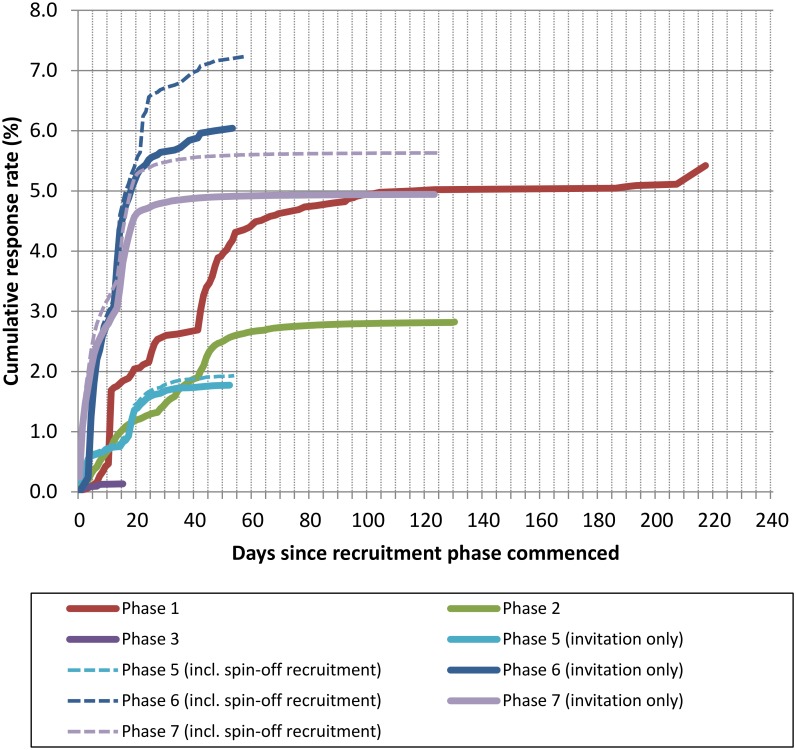
Cumulative response rates to UK COSMOS study invitations, by recruitment phase, 2009–2012. Fig 1 Footnotes: Phase 1 used a mobile phone subscriber sampling frame, letter invitation, paper consent and registration, questionnaire via paper or web and no incentive. Phase 2 used a mobile phone subscriber sampling frame, letter invitation, web-based consent, registration, and questionnaire and a prize draw incentive. Phase 3 used a direct marketing list sampling frame, SMS invitation, web-based consent, registration, and questionnaire and no incentive. Phase 5 used an electoral register sampling frame, letter invitation, web-based consent, registration, and questionnaire and a prize draw incentive. Phase 6 used an electoral register sampling frame, letter invitation, web-based consent, registration, and questionnaire and a gift voucher incentive. Phase 7 used an electoral register sampling frame, letter invitation, web-based consent, registration, and questionnaire and a gift voucher incentive. ‘Invitation only’ represents recruitment of invitee named on letter, and ‘Spin-off recruitment’ represents recruitment of additional friends and family.

In our first large-scale recruitment campaign using the fully web-based system (Phase 2), we invited ~2.4 million people and achieved rapid recruitment (~60,000 participants in 7 weeks) and a response rate of 2.8%. Comparing Phases 1 and 2 (Table 1) there was a clear penalty of using a web-only process in terms of decreased response rate—probably reflecting lost ‘paper responders’. However, cost-efficiency was vastly improved in Phase 2, with cost per participant recruited being ~16% of that in Phase 1. Using a web-based system speeded up recruitment and improved efficiency. Peak recruitment and baseline data collection was achieved very quickly, within 30–60 days (Fig 1). Acceptability and accessibility of new methods to participants is critical to success. We experienced very few adverse reactions (N = 15 irate people, i.e. <0.01%) during Phase 2, and these related to how individuals had been identified for mailing not the use of web-based methods. In Phase 2, our first large-scale recruitment campaign, 7890 calls were received by the call centre in the first 8 weeks of recruitment, averaging 164 calls per day, with a peak of 695 calls in a single day.

Our web-based system streamlined registration, consent and questionnaire into one process. The mixed paper-web system (Phase 1) resulted in a higher proportion of participants without any questionnaire data compared to web-only (Phase 2) (22.5% vs 4.9% respectively), reflecting non-return of paper questionnaires. Use of paper consent forms separated consent and questionnaire processes in Phase 1, producing inconsistency i.e. completion of only one of consent or questionnaire, which reduced participant value and involved time-consuming follow-up of missing forms.

We found response rates to SMS invitations to be very low: 0.11% for Group 1 (initial SMS followed by reminder SMS) and 0.18% for Group 2 (initial SMS followed by reminder e-mail). Cost per participant recruited using SMS invitation (incorporating data rental/ SMS/ email broadcast) was 18 times higher than cost per participant for printing/mailing in Phase 2. We observed that message delivery failure was higher for SMS (9.2%) compared to email (5.5%).

Our study invitation advert on Facebook was shown 1,841,684 times during the 4 weeks it was live, and had a click-through rate of 0.03%. This resulted in 236 new visitors to our study website and 2 new study participants. As the per participant cost of the Facebook trial far exceeded that for letter invitations (although less than for SMS invitations) we did not pursue this further.

We reverted to invitation by letter for subsequent recruitment, but reduced printing and mailing costs by directing potential participants to the study information leaflet available on our website, rather than including a printed version with the letter.

We observed that a paid incentive of a £10 gift voucher on questionnaire completion more than tripled response rate (6.0% of those sent invitations) in a Phase 6 pilot study compared to the 1.9% response rate achieved using a prize draw incentive (20 x £100 prizes) in Phase 5 (Table 1, Fig 1). We used the £10 paid incentive for further large-scale recruitment (Phase 7) with invitation letters to 645,000 people. The overall response rate was 5.6% with >34,000 new participants recruited in just 3 weeks. Per-participant recruitment cost was ~10% lower compared to our first large-scale campaign (comparing spend on printing, mailing and incentives in Phases 2 and 7), and ~35% lower than projected cost of scaling up Phase 5 which used comparable sampling frame with a prize draw incentive.

In Phase 1, a reminder invitation letter was posted to non-responders on Day 40, by which time responses had begun to plateau. The reminder approximately doubled the response rate (Fig 1), and as a result we used postal reminders in all subsequent phases.

We used email and SMS reminders to encourage questionnaire completion amongst participants who had not completed in a single web session. Following 2 email reminders, an SMS reminder appeared to be more effective than another email reminder (completion rates 18.9% and 7.1% respectively). We also observed that an email reminder including a deadline resulted in a higher completion rate than one without (completion rates 18.7% and 7.1% respectively).

Taking into account unsubscribes, contact preferences, and email bounces we were able to send an e-newsletter to 96% (N = 100,844) of our cohort in 2014. In 2011 we collected information on email delivery success, opening and click-throughs when we sent out our e-newsletter, and observed that 2% of emails could not be delivered (N = 1405 emails bounced out of 66063), and achieved e-mail open and click-through rates of 34% (N = 22,317) and 14% (N = 9,447) respectively. In response to our annual email request asking participants to confirm/update their details, typically 18–19% do so, with 27% (N = 27,811) of the cohort ever updating details via the portal.

**Fig 2:**
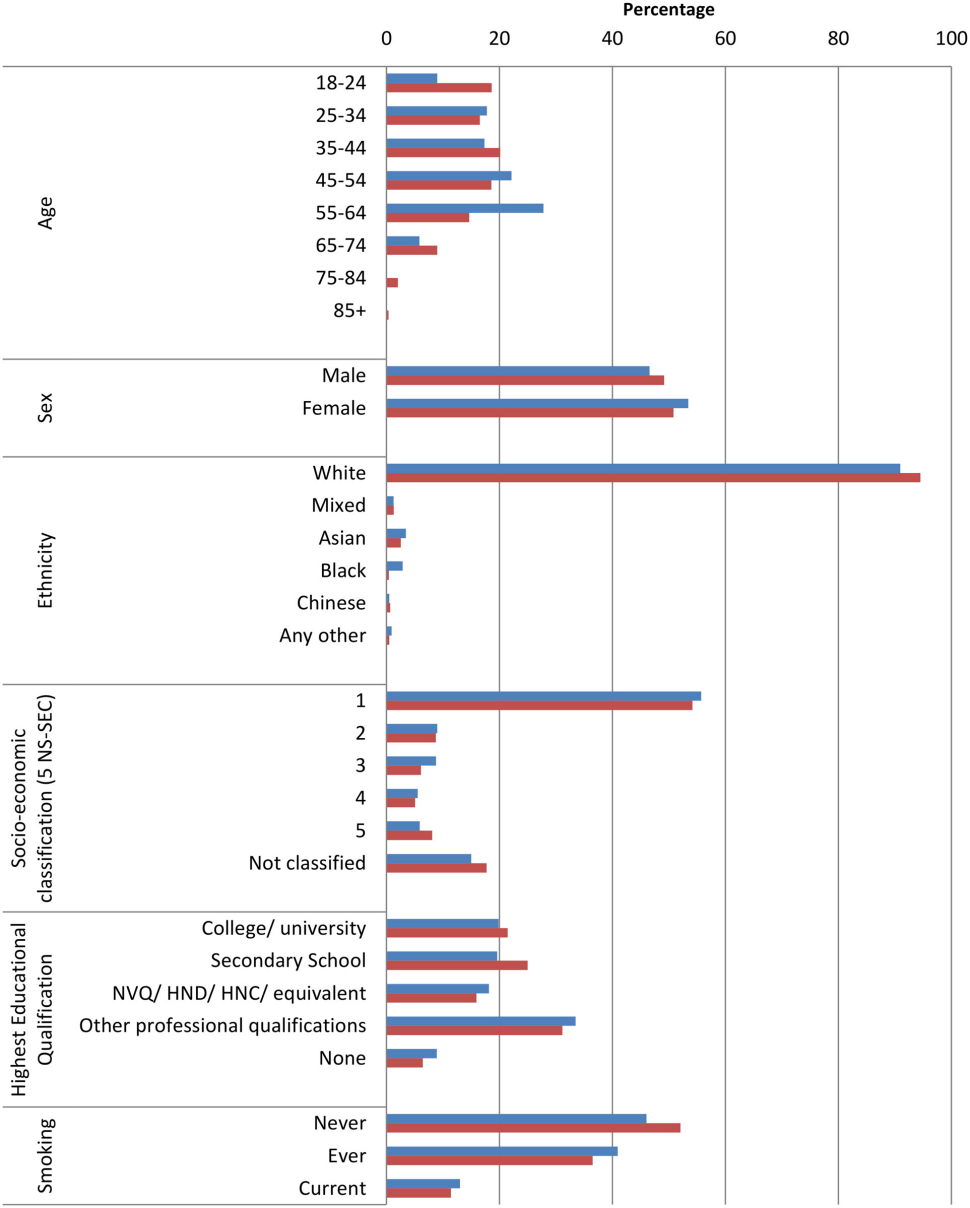
Characteristics of participants from two major recruitment campaigns to the UK COSMOS study (Phases 2 and 7). Legend: Blue bars represent Phase 2, red bars represent Phase 7. Fig 2 Footnotes: Phase 2 used a mobile phone subscriber sampling frame, letter invitation, web-based consent, registration, and questionnaire and a prize draw incentive, and recruited N = 67,793. Phase 7 used an electoral register sampling frame, letter invitation, web-based consent, registration, and questionnaire and a gift voucher incentive, and recruited N = 36,316. Together Phases 2 and 7 recruited N = 104,109. The profile of participants presented here is based on N = 67627 from Phase 2 and N = 36218 from Phase 7, i.e. excluding 264 withdrawals. With the exception of socio-economic classification, the percentages calculated exclude Missing from the denominator. N for missing are as follows: Phase 2: Sex N = 290, Age group N = 306, Ethnicity N = 9205, Highest Educational Qualification N = 9124, Smoking N = 8404; Phase 7: Sex N = 2, Age group N = 9, Ethnicity N = 5135, Highest Educational Qualification N = 5083, Smoking N = 4760. For socio-economic classification Missing are included in the Not classified category, which also contains people who never worked or were long-term unemployed and therefore could not be assigned a classification based on occupation.

Sex, age, ethnic, socio-economic status (SES) and smoking distributions were broadly similar for our two major recruitment campaigns, Phases 2 and 7 (Fig 2). The main difference was for age, with slightly lower mean age in Phase 7 vs Phase 2 (43 and 46 years respectively). Participants in both campaigns were predominantly White, with two-thirds from the highest social class, and highly educated (53% have degree and/or professional qualifications).

Based on our experiences we set out our recommendations for methods to establish and follow-up a large prospective cohort, and tips for using these methods, in Table 2.

**Table 2:** Recommendations for How to Establish and Follow-up a Large Prospective Cohort.

Methods at each stage	Recommended	Tips
**1. Choice of sampling population**		
Direct marketing list	?	- Use a reputable supplier, e.g. abiding by the Direct Marketing Code of Practice in the UK.
		- Ask how the list has been compiled, e.g. sources used, how people on the list opted-in?
Edited Electoral Register	Yes	- Commercial suppliers hold compiled lists for the UK—avoids dealing with multiple councils.
		- May not be fully representative of the base population.
**2. Invitation method**		
Letter	Yes	- Large-scale mailing is cheaper and more efficient through a commercial mailing house.
SMS and email	No	
Facebook/Social media	?	- Test a variety of adverts/invitations.
		- Use Facebook performance tracking to evaluate and optimize your advertising criteria [36].
		- Adjust advertising campaign hours and your cost-per-click bids to take advantage of your target population’s Facebook routines [37].
		- Set up a Facebook page about the study and who is conducting it to increase the study's credibility with your target audience [15].
**3. Data collection**		
Paper	No	
Fully web-based process for consent, recruitment and data collection	Yes	- Seek expert advice on security of your web-based process.
		- Test web-based systems extensively before ‘going live’.
		- No physical copies of the data exist—reliable data back-up is essential.
		- Ensure your system can deal with high levels of web traffic.
		- Have sufficient resources, e.g. call centre, technical back-up, in place to resolve problems quickly if they arise—to maintain reputation and avoid loss of participants.
		- Good signposting of progress through a web-questionnaire, particularly if it is long.
		- Choose survey software carefully: some are inflexible re question types, and questions designed on paper may be difficult to convert. Develop your questionnaire with known software in mind.
		- Use a web statistics service, e.g. Google Analytics, to evaluate your website traffic and use this to improve your website/system.
		- Build in checks (e.g. multiple entries from single IP address/with same email address) to identify duplicates and poor quality data, and to prevent abuse of any incentive offered.
**4. Increasing response rate**		
Prize draw	?	
Paid incentive, e.g. gift voucher	Yes	- State Terms and Conditions (T&Cs) clearly to manage participant expectations.
		- Set a limit on number of vouchers you will supply and a deadline for the offer.
		- Arrange automatic email delivery of vouchers by voucher supplier to simplify logistics.
		- Allow sufficient timeframe within T&Cs to supply vouchers to participants.
Spin-off recruitment, e.g. via family and friends	Yes	- May not be representative.
**5. Using reminders…**		
**…to increase response rate**		
Letter	Yes	- To minimise the number sent and save costs, monitor response rates in real-time, and wait until recruitment begins to plateau before sending a reminder.
		- Include a statement that it is a reminder within the letter.
**…for data collection**		
Email	Yes	- Record number of recipients opening email, useful for evaluation.
		- In email content/subject line avoid key words/phrases which trigger spam filters.
		- Good practice to include unsubscribe option for emails, and essential if using a commercial provider for email broadcast.
		- Make clear that unsubscribing is not the same as withdrawing from study.
		- Include deadline.
SMS	Yes	- Use to reach those who don’t respond to email reminders.
		- Include deadline.
**6. Direct follow-up with participants**		
Email	Yes	- See reminder email above.
Facebook/Social media	Yes	- Capture group members and followers at recruitment whilst interest is fresh.
		- Maintain an active social media presence to keep participants engaged.

## Discussion

### Choice of sampling population

Sampling from mobile subscriber lists was specific to our cohort, but appropriate given our research question. Our experience of using direct marketing data for recruitment via SMS/email was not successful but that may reflect invitation method rather than data source. Another environmental epidemiology study reports successfully using direct marketing lists for recruitment by letter/telephone [8]. Whilst not suitable for very specific cohort studies (e.g. birth cohorts, or for investigating occupational exposures), we recommend that researchers consider general population sampling from commercially available mailing lists/electoral register lists, which can offer millions of records, when collecting questionnaire information from the general adult population and where invitation is by letter (Table 2).

Choice of sampling population is dependent on the research question and invitation method. Large data sources are essential to provide sufficient people to invite in order to obtain the required sample size. Looking to the future, the increasing volume of data being collected by organisations such as Facebook and Google allows ever more detailed individual profiling—the huge commercial value of these data for targeted marketing is already recognised, but they are also valuable as a potential resource for research. The ability to identify specific study populations, e.g. night shift workers from timing of online activity or those with particular diets from their online shopping data, would make targeted study invitations possible and offer a new way of identifying and inviting study populations for health research, provided that any concerns over data privacy are addressed.

Data can be purchased for cold calling purposes if individuals have indicated that they are willing to receive third party mailings/communications, typically by ticking or unticking the relevant box when they are providing their personal data. Researchers can purchase these data from companies which offer such data, and use these to directly approach potential study participants via a variety of contact routes, as we did via SMS and email, depending on the contact preferences individuals have indicated. Whilst anyone can purchase and use these data for cold calling, be it for commercial marketing purposes or non-commercial purposes such as research recruitment, they still need to abide by data protection laws whilst doing so and this could include allowing the recipient to opt-out from further communications, or a limit on the number of communications. For example, in the UK, researchers would need to comply with the Data Protection Act 1998, and if contact was to be by email or SMS they would additionally need to comply with the Privacy and Electronic Communications (EC Directive) Regulations 2003. We suggest that if researchers are unsure about the legal requirements, that they seek guidance from the Information Commissioner’s Office (in the UK) or equivalent body in their country to ensure that data use is in accordance with local legal regulations.

### Invitation method

SMS text messaging has been used successfully in healthcare practice and epidemiological research [9–11]. To our knowledge, however, SMS has never been used for ‘first contact’ to invite people to participate in health research. SMS invitations were not cost effective due to very low response rate, and we would not recommend SMS for ‘first contact’ with potential study participants (Table 2). The low response rate may reflect a combination of restricted content (160 character limit), lack of formality/difficulty in demonstrating bona fides, limited brand recognition, possible fatigue associated with SMS/e-mail marketing communications, and quality of direct marketing data.

We would therefore still recommend invitations by letter due to higher response rate and lower overall cost (Table 2). However, our finding does not mean that invitations sent to mobile phones would never work. Multimedia messaging service (MMS) and instant messaging apps, e.g. Whatsapp or BlackBerry Messenger, are versatile—they have no character limit and can embed pictures/videos. Embedding a formal invitation letter as an image might demonstrate bona fides and overcome the informality of SMS, whilst video content could provide engaging study information. Recipients would need a smartphone to benefit from rich content and to follow through to a study website immediately. Smartphone uptake is rapid (61% in UK [12] and 58% in US [13]), so should not be a barrier in future. Bulk MMS is possible, but we did not test this due to higher costs. If free messaging apps could be utilised this would reduce costs. This invitation method would be limited to those with the app already installed, and would rely on ability to identify app users, those users accepting the Sender as a contact (BlackBerry Messenger only), and the ability to bulk send (currently ‘group chats’ on Whatsapp limited to 50) but has potential to reach some people at very low cost. Ultimately, whether such an approach was appropriate would depend upon the research question and study population.

Social networking sites (SNS) are potential tools for cohort recruitment and direct follow-up with participants, with a wide reach (e.g. 829 million daily Facebook users [14]). There are various Facebook recruitment methods: paid advertising, Facebook searches, Facebook posting and snowball sampling [15]. Paid for targeted study adverts can be displayed on Facebook, e.g. by selecting on age, sex, location, and keywords in personal profiles. Consistent with our experience, others similarly found that click-through rates are low (<0.1%) with participation rates amongst click-throughs varying widely (<1 to 10%) [16, 17], and response differing by advert where several were trialled. To date, evidence re cost-effectiveness of recruitment using paid Facebook advertising is mixed [15]. Potential limitations of SNS recruitment include the inability to randomly sample SNS users which may bias samples; in particular, variation in SNS use by age and gender may result in underrepresentation of males and older individuals [15]. We do not currently consider large-scale cohort recruitment to be feasible by SNS recruitment alone. However, it may be useful for boosting recruitment amongst particular demographic groups, such as younger age groups.

Response rates were very low for all invitation methods used, when comparing against large-scale epidemiology studies conducted in the past [2–4]. However, they are on par with the 5.5% response rate achieved in recent years by UK Biobank [5]. This begs the question what is an acceptable response rate in the 21^st^ Century? Recent editorials and commentaries note that a 60% response rate has long been used as the threshold of acceptability, and is a minimum required by some biomedical journals, but that it is a rule of thumb without a firm statistical basis and that there is no scientifically proven minimally acceptable response rate [18, 19]. There is a fixation on increasing response rates as a proxy for reducing nonresponse bias, but it is noted that response rate may not be as strongly associated with study quality, representativeness or bias as is often believed, and it is suggested that researchers should consider whether funds allocated to maximizing response rates might be better spent on nonresponse bias analyses or increasing the effective sample size of a study [18, 20].

### Data collection

Internet access increases year on year making web-based research widely accessible (84% of households in Great Britain have internet access [21]). Outside research, people regularly give consent for confidential data to be used online, e.g. online banking and credit card billing. At the time of designing our system a number of epidemiological/health-related studies had or were using web-based consent procedures (e.g. [22, 23]), but we could find only one long-term cohort study amongst these [24]. Some required an electronic ‘signature’ and some gave the option of paper consent and questionnaire, and those intending to access health records still required hard-copy signed medical release forms. To our knowledge, ours is the earliest use of remote web-based e-consent (without signature) for health research with access to, and long term follow-up of, health records within the UK. Since then various studies, online health programmes and online disease registries (e.g. [25–28]) report using online consent procedures, although often little detail is given as to what exactly this entailed.

Person verification was the main concern of web-based signatureless e-consent, given that such consent would be used to access confidential data. However, a signature is no longer the gold-standard for personal identification, as with the example of secure financial transactions. Personal cheque use is in terminal decline, and security of card payments has been improved by use of chip and PIN, in place of signing a receipt. Our registration procedure reduced the possibility of identity fraud, as it required provision of data (e.g. date of birth, mobile phone number) which are a) not given in the invitation letter and b) must correctly match name and address held by other data providers, in order to access and follow-up health records and/or mobile traffic data.

We achieved very rapid recruitment via a web-based system, but the inherent risk with this approach is that any technical glitch could have a large impact in terms of loss of participants or data. Extensive error testing is required prior to going live, and resources in place to deal with high volumes of technical and other queries from participants. We received a broad range of queries, but some of the most common related to how people had been identified to receive a study invitation and/or how Imperial College had accessed their data; how data collected in the study would be used; when study results would be available; whether it was worth individuals who used a mobile phone only a little or not at all joining the study; technical issues with the web-based system, e.g. having difficulty logging in; requesting clarification of specific questions in the questionnaire; receipt of gift vouchers (where these were offered); and wanting to provide additional information regarding health or mobile phone use that the participant was not asked for in the questionnaire.

Unless a face-to-face interview is required at recruitment (e.g. for immediate biospecimen collection or assessment of physical measures at recruitment), we would strongly recommend web-based consent and data collection by the participant at home (Table 2). Biospecimens could still be collected later, or by post (e.g. saliva), and time/costs associated with clinic visits reduced. Although this might result in an increased proportion with missing biospecimens, overall a larger cohort might be recruited because completing consent and questionnaires at home is more convenient, especially for people of working age.

### Use of incentives

Automatic email delivery by our voucher supplier made voucher distribution easy and efficient. However, spam filters or email send errors can mean that some vouchers do not reach participants first time, and resources need to be in place to deal with resulting enquiries. We cannot yet assess whether recruitment using a paid incentive affects long-term commitment to the research. However, the current study withdrawal rate is similar for those receiving vouchers (0.3%) vs. those to whom a voucher was not offered (0.3%). Motivation to complete follow-up questionnaires may differ according to expectations generated by recruitment incentives, and this will need to be evaluated in the future.

### Using reminders

If a respondent loses interest during recruitment/data collection reengaging them may be difficult and costly. The benefits of using email and SMS for reminders are that they can be automated, provide click-through links to study websites and questionnaires and are relatively inexpensive, compared to telephone or mail. We recommend using email and SMS in combination to achieve maximum effect (Table 2).

### Direct follow-up with participants

Ongoing participant engagement and maintaining up to date participant details are critical to long-term cohort follow-up, particularly where this relies on participants completing follow-up questionnaires. We are able to send e-newsletters to the vast majority of our study participants, and achieve e-mail open and click-through rates which are well above those in the e-mail marketing industry (e.g. 22.87% and 3.26% respectively [29]). It is useful to compare with the experience of UK Biobank, where annual newsletters are emailed to all participants for whom an email address is available (N = 320,183 approximately 64%), but by post to approximately 182,000 others and where the email bounces (N = 9055 in 2014). UK Biobank participants are encouraged to update details/provide an email address at every opportunity, and email address provision is climbing (up from 294,834 in 2012)(A Trehearne, UK Biobank, personal communication, 2014).

Email will not reach everyone, but used as a first step it will reduce the number to be contacted by phone/mail thus reducing costs, and may reach participants who have moved address and would otherwise be lost to follow-up. We recommend collecting email address and mobile number as standard at registration, and subsequently, to allow low-cost routes for future contact.

Retention and follow-up of participants in longitudinal studies may be improved by using social media, e.g. participants could be invited to join a Facebook group and/or follow a Twitter feed. We recommend integrating social media at the outset in order to capture group members and followers at recruitment whilst their interest is fresh (Table 2). It costs nothing to use, but time is required to generate content, post news and updates. Spin-off recruitment amongst participants’ social networks may be an additional benefit, if the research question/study population reflects a specific shared experience or interest, e.g. a birth cohort, occupational cohort, or a study located in a specific geographical area, which is likely to be reflected within participants’ social networks. Studies have used Facebook to identify and contact study participants not responding to other contact methods, thus reducing loss to follow-up [30, 31].

### Do the recruitment methods used influence who participates?

It is important for researchers to consider whether their chosen recruitment methods may introduce potential biases or affect the representativeness of their cohort. For example, in UK COSMOS, because we recruited from a mobile subscriber sample, we could have excluded people such as migrants or temporary residents who may be more likely to use pay-as-you-go rather than have a mobile phone subscription. Likewise, migrants or temporary residents who are not eligible to vote in the UK will not be on the Edited Electoral Register, which we also used as a sampling frame. Participants in our cohort are predominantly White, with a large proportion being from the highest social class, and highly educated. Whilst this reflects the majority White ethnicity of the UK population (86% in England & Wales [32]), and that wealthier, better educated people are more likely to participate in research, it is also possible that our sampling frames may have influenced our final cohort profile, and thus the representativeness of our study population. In cohort studies, bias resulting from differential selection at start of follow-up is a form of confounding which can be controlled for by adjusting for the factors responsible for selection differences [33]. True selection bias arises from selection affected by the exposure under study, for example if loss to follow-up is associated with both the exposure and risk of the outcome [33, 34].

Aside from the influence of specific recruitment methods, researchers should also consider that representativeness will be influenced by the type of people who participate in research having a different profile from the general population. Low response rates may reflect research fatigue resulting from people being bombarded with survey requests nowadays, and apathy to research with research participation only really appealing to those who understand the benefit. As those participating in research tend to be more affluent and better educated, this could result in a healthy cohort effect, i.e. a cohort with lower disease incidence and higher disease survival rates compared to the general population. Thus, effect estimates may not be fully generalizable to the wider population, but should not be biased as long as nonparticipation is not associated with exposure, and factors related to selection are controlled for in the analysis. As participants might have lower levels of general risk factors for adverse health outcomes (e.g. alcohol, smoking, and poor diet), there may be less potential for residual confounding by these general risk factors, making it easier to tease out underlying exposure-response relationships.

Additionally, researchers should consider whether their particular research focus could influence participation. For example, in UK COSMOS, we considered whether people interested in our research question (use of mobile phones, wireless technologies and health), were also likely to be more amenable to web-based participation methods. Overall we think not, as mobile phone use is now so commonplace (93% of UK adults [12]) that the underlying sampling population is unlikely to be biased.

## Conclusions

Web-based and mobile phone technologies make it possible to set up and follow up large cohorts with greater logistical ease and significant cost and time savings compared with traditional methods. In particular, data collection is streamlined, recruitment progress can be tracked automatically, and data can be automatically coded improving data integrity.

We fully recommend the use of web-based consent, recruitment and data collection, where this is appropriate. However, you need to be selective in your choice of technologies and what task you use them for—as we have found, not all will be successful, and may work for one task but not others. You also need to keep up to speed with technological changes and trends, e.g. web-based recruitment and data collection should be optimised for tablets and smartphones with touch-screen technology, as their market share grows. Any recruitment campaign and web-based data collection system needs to look appealing, be slick and accessible on all devices to ensure success. Reminders are a valuable tool to boost participation rates and to encourage completion of web-based questionnaires.

Large-scale cohort studies are invaluable in epidemiology but are expensive to set up and maintain [35]. They can be set up more cost-effectively and more quickly by harnessing new technology. But to be successful you have to understand how and when to use these technologies. We hope that our experience and lessons learnt during the UK COSMOS study can help future researchers achieve this.

## Supporting Information

S1 File Supporting Information. This file contains aggregated count data underlying Figs 1 and 2 and findings regarding questionnaire completion, SMS and email delivery, SMS invitation response rates, and Facebook invitation response rates referred to in the text of the manuscript.
(XLSX)
